# Delineating Novel and Known Pathogenic Variants in *TYR*, *OCA2* and *HPS-1* Genes in Eight Oculocutaneous Albinism (OCA) Pakistani Families

**DOI:** 10.3390/genes13030503

**Published:** 2022-03-12

**Authors:** Muhammad Shakil, Abida Akbar, Nazish Mahmood Aisha, Intzar Hussain, Muhammad Ikram Ullah, Muhammad Atif, Haiba Kaul, Ali Amar, Muhammad Zahid Latif, Muhammad Atif Qureshi, Saqib Mahmood

**Affiliations:** 1 Department of Biochemistry, University of Health Sciences (UHS), Lahore 54600, Pakistan; shakil757@gmail.com; 2 Department of Biochemistry, King Edward Medical University, Lahore 54000, Pakistan; 3 Department of Biological Sciences, International Islamic University, Islamabad 44000, Pakistan; abida.phdbt24@iiu.edu.pk; 4 Department of Biochemistry, Services Institute of Medical Sciences, Lahore 54600, Pakistan; haidernazish144@gmail.com; 5 Department of Ophthalmology Services, Institute of Medical Sciences, Lahore 54600, Pakistan; intzardr@hotmail.com; 6 Department of Clinical Laboratory Sciences, College of Applied Medical Sciences, Jouf University, Sakaka 75471, Saudi Arabia; mikramullah@ju.edu.sa (M.I.U.); maahmad@ju.edu.sa (M.A.); 7 Genetics Discipline, Department of Animal Breeding and Genetics, Faculty of Animal Production and Technology, Ravi Campus, University of Veterinary and Animal Sciences, Pattoki 55300, Pakistan; haibakaul@gmail.com; 8 Department of Human Genetics and Molecular Biology, University of Health Sciences, Lahore 54600, Pakistan; ali.amar@uhs.edu.pk; 9 Department of Community Medicine and Medical Education, Azra Naheed Medical College, The Superior University, Lahore 54600, Pakistan; mzahidlatif@yahoo.com (M.Z.L.); matifqureshi1@gmail.com (M.A.Q.); 10 Institute of Biomedical & Allied Health Sciences, University of Health Sciences Lahore, Lahore 54600, Pakistan

**Keywords:** oculocutaneous albinism (OCA), Pakistani families, pathogenic variants, *TYR*, *OCA2*, *HSP*

## Abstract

Oculocutaneous albinism (OCA) is associated with a wide range of clinical presentations and has been categorized with syndromic and non-syndromic features. The most common causative genes in non-syndromic OCA are *TYR* and *OCA2* and *HSP1* is in the syndromic albinism. The objective of this study was to identify pathogenic variants in congenital OCA families from Pakistan. Eight consanguineous families were recruited, and clinical and ophthalmological examination was carried out to diagnose the disease. Whole blood was collected from the participating individuals, and genomic DNA was extracted for sequencing analysis. TruSight one-panel sequencing was carried out on one affected individual of each family, and termination Sanger sequencing was carried out to establish the co-segregation of the causative gene or genes. In silico analysis was conducted to predict the causative pathogenic variants. Two families were found to have novel genetic pathogenic variants, and six families harbored previously reported variants. One novel compound heterozygous pathogenic variant in the *TYR* gene, c.1002delA; p.Ala335LeufsTer20, a novel frameshift deletion pathogenic variant and c.832C>T; and p.Arg278Ter (a known pathogenic variant) were found in one family, whereas *HPS1*; c.437G>A; and p.Trp146Ter were detected in another family. The identification of new and previous pathogenic variants in *TYR*, *OCA2*, and *HPS1* genes are causative of congenital OCA, and these findings are expanding the heterogeneity of OCA.

## 1. Introduction

Albinism is a rare genetic disorder arising from the impairment of melanin biosynthesis. It is categorized into ocular albinism, which influences only the eyes; and oculocutaneous albinism, which involves the skin, eyes, and hair. Oculocutaneous albinism (OCA) is a rare genetic anomaly consequence due to the complete melanin deficiency or partial or reduced synthesis of pigment in eyes, hair, and skin. OCA-affected individuals have relatively fair complexions compared to their unaffected relatives [1]. The clinical presentations of OCA are variable in each subtype. These features include nystagmus, photophobia, hypopigmentation of skin, hair and eyes, mild to moderate reduced visual acuity, and iridis trans-illumination [2,3,4]. OCA is further divided into syndromic and non-syndromic OCA (nsOCA) types. As of now, eighteen OCA-associated genes have been reported in syndromic and non-syndromic phenotypes. Out of these genes, seven genes/loci are linked to autosomal recessive nsOCA. These genes include *TYR*, *OCA2*, *TYRP1*, *SLC45A2*, *SLC24A5*, *C10orf11*, and *DCT* [1,2,3,4]. These genes are subsequently related to type OCA1-8, except the OCA5 locus, which has been described in OCA families of Pakistani origin. The causative gene has not yet been detected [5]. The recently identified type OCA8 is linked to *DCT,* as reported in Turkish and French populations [3,4]. Worldwide, more than 400 pathogenic variants have been identified in *TYR,* and more than 300 pathogenic variants are associated with *OCA2*. The most commonly reported subtype is OCA1 in the Caucasian population, and about 50% of cases are documented worldwide [6,7]. In Pakistani families, OCA1 is the most common OCA type, followed by OCA2 [1,2,8,9].

On the other hand, Hermansky–Pudlak syndrome (HPS) is one of the syndromes associated with albinism. It is a multisystem autosomal recessive syndrome characterized by OCA, excessive bleeding, and, in some individuals, fatal pulmonary fibrosis, granulomatous colitis, and immunodeficiency. The associated ocular abnormalities include nystagmus and foveal hypoplasia with a substantial reduction in visual acuity [10]. Eleven different HPS types have been reported (HPS1–HPS-11) with common visual acuity and variable bleeding manifestations [11,12,13]. HPS-1 affects individuals of different ethnicities, including those from Western Europe, Japan, the Middle East, the Indian Subcontinent, and non-Puerto Rican Hispanic people [14,15,16]. HPS affects 1/1,500,000–1,000,000 individuals globally, although the prevalence is 1/1800 in individuals of Puerto Rican origin [17]. The group of BLOC-2 including HPS-3 and HPS-5&6 contains milder and quite rare subtypes [18]. The group of BLOC-1 including HPS-7, HPS-8, and HPS-9 contains extremely rare subtypes [19].

In the present study, we have identified the consanguineous OCA families’ pathogenic variant of the *TYR*, *OCA2*, and *HPS-1* genes in the Pakistani population.

## 2. Patients and Methods

### 2.1. Detail of Families

Ethical approval for this research was obtained from the Ethical Review Committee (ERC) of University of Health Sciences Lahore, and the human subjects were included according to the Helsinki guidelines (2008). This study was carried out on eight OCA Pakistani families. All the congenital families originated and were recruited from the province of the Punjab with distinct ethnic backgrounds; 1—Mayo (Lahore), 2—Cheema (Sargodha), 3—Rajpoot (Rawalpindi), 4—Arain (Sheikhupora), 5—Dogar (Burewala), 6—Dogar (Kasur), 7—Bhatti Rajpoot (Lahore), 8—Bukhari Sayyed (Rahim Yar Khan). To establish the diagnosis of a pro-band in each family, familial history and clinical presentation were assisted by clinicians/ophthalmologists from the regional teaching hospitals. The phenotype was examined by physical inspection of skin, hair, and eye color. Clinical images/photos of the individuals affected with OCA were taken to confirm the disease pattern. Ophthalmic examinations were carried out to assess the visual acuity and color vision testing, and direct ophthalmoscopy was used for fundoscopic examination. These findings were documented on data forms.

### 2.2. Molecular Genetic Analysis

An ethylenediaminetetraacetic acid (EDTA) blood sample was collected from each participating individual, and genomic DNA extraction was carried out as previously described [8]. One affected individual of every family underwent TruSight gene panel sequencing, which contained all the syndromic and non-syndromic variants associated with albinism. Then, Sanger sequencing carried out the co-segregation of pathogenic variants in putative albinism genes (*TYR*, *OCA2*, and *HPS1*) in respective families. All the data was aligned to the human genome reference sequence (hg19) to observe sequence variants using BioEdit 7.0 software (http://www.mbio.ncsu.edu/BioEdit/bioedit.html, accessed on 27 August 2019), Chromas Lite (http://technelysium.com.au/wp/chromas/, accessed on 27 August 2019) software, and CLC sequence viewer (https://www.qiagenbioinformatics.com/products/clc-sequence-viewer/, accessed on 27 August 2019).

The in silico tools applied to predict the pathogenicity were PolyPhen2, SIFT, and Pathogenic variant Taster [20]. For the missense pathogenic variants, multiple sequence alignment (MSA) determined the evolutionary conservation of mutated amino acid residues in TYR and OCA2 orthologs by using MEGA-X software, Pennsylvania State University (https://www.megasoftware.net/, accessed on 21 December 2020). In the absence of an experimentally determined crystal protein structure, the SWISS-MODEL server (https://swissmodel.expasy.org/, accessed on 21 December 2020) was used for homology protein modelling of wild-type TYR protein. Subsequently, 3D protein modelling analysis and visualization of the *TYR* p.Arg278Ter and p.Ala335LeufsTer20 pathogenic variants were performed using PyMOL software (http://www.pymol.org, accessed on 17 November 2021).

## 3. Results

### 3.1. Clinical Presentation of OCA Cases

A total of eight OCA families were recruited and investigated for pathogenic variant analysis. Inherent and pedigree analysis established an autosomal recessive mode of inheritance in all families, and each affected individual of the family presented cardinal features consistent with OCA. These characteristics included pale to reddish-white skin, white to golden blonde hair, photophobia, nystagmus, reduced visual acuity of variable degree, and strabismus. In OCA families, the ophthalmological examinations showed the classical characteristics of albinism, such as nystagmus, foveal hypoplasia (moderate degree), hypo-pigmented fundus, and strabismus. A summary of clinical findings observed in OCA families is represented in Table 1.

### 3.2. Genetic Pathogenic Variants

#### 3.2.1. Novel and Known Pathogenic Variants in *TYR* Gene

**Figure 1:**
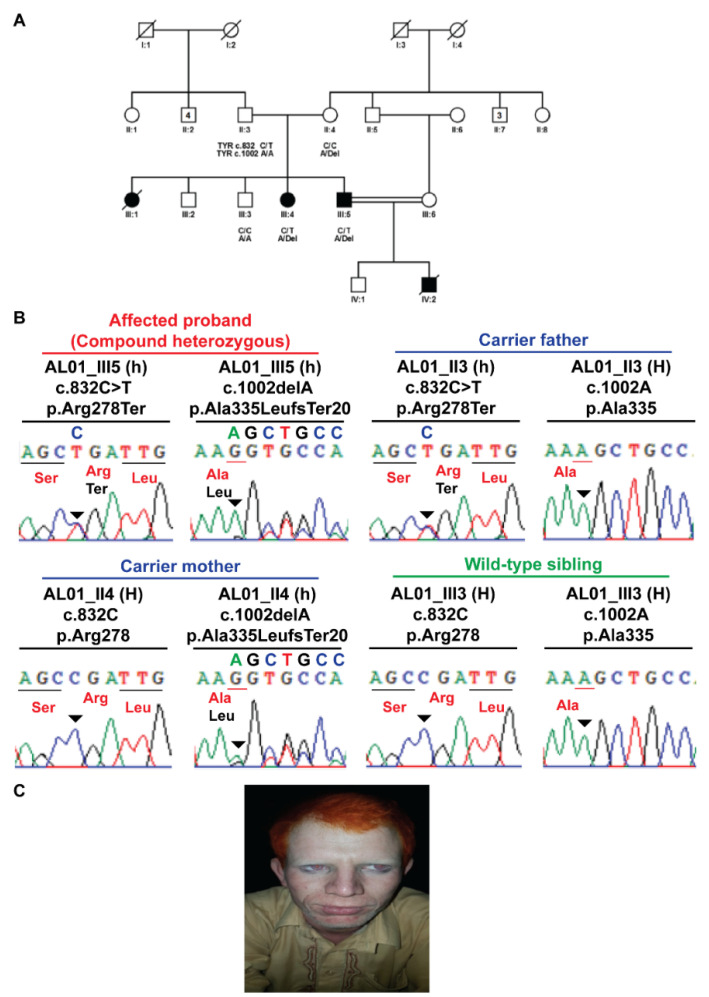
Compound heterozygous *TYR* p.Arg278Ter and p.Ala335LeufsTer20 variants in AL01 family manifesting albinism. (**A**) Pedigree structure of AL01 family. (**B**) Sanger sequencing electropherograms reflecting familial segregation of *TYR* p.Arg278Ter and p.Ala335LeufsTer20 pathogenic variants in AL01 family. (**C**) Clinical image depicting clinical features of albinism in pro-band III-5 of family AL01.

In albinism family number 01 (AL01), the combination of sequencing techniques determined a novel compound heterozygous pathogenic variant in the *TYR* gene in which one allele c.832C>T; p.Arg278Ter was a previously known pathogenic variant, whereas another pathogenic variant c.1002delA; p.Ala335LeufsTer20 was a novel frameshift deletion. This is the native compound heterozygous pathogenic variant associated with the OCA phenotype. In co-segregation analysis, the father was a homozygous wild type (WT) for c.1002delA and a heterozygous carrier for allele c.832C>T, whereas the mother was homozygous WT for c.832C>T and heterozygous for c.1002delA variant. Both the affected members showed heterozygous alleles, one c.832C>T contributed by the father and another allele c.1002delA received from the mother to produce the OCA phenotype, as seen in Figure 1A–C.

**Figure 2:**
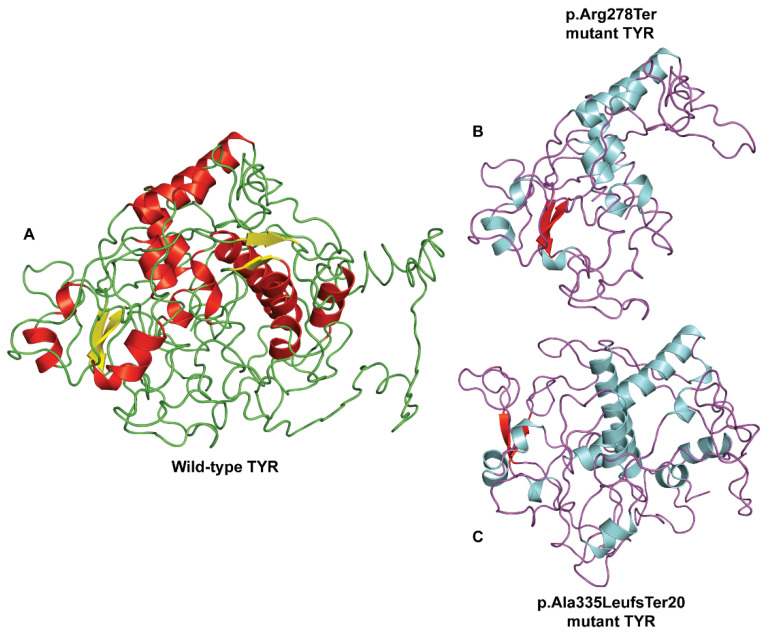
The 3D protein modelling of p.Arg278Ter and p.Ala335LeufsTer20 pathogenic variants in the predicted crystal structure of human TYR protein. (**A**) Wild-type protein structure of TYR protein as predicted using SWISS-MODEL. Comparison of the wild-type and mutant TYR protein structures reveals loss of secondary structure elements and changes in the overall 3D protein structure for truncating (**B**) p.Arg278Ter and (**C**) p.Ala335LeufsTer20 pathogenic variants.

An in silico analysis was carried out to explore the relationship between the mutant TYR and the wild-type protein (Figure 2A). It was predicted that a nonsense mutant allele of p.Arg278Ter may result in early termination and causes premature truncation in the TYR protein of 251 amino acids only (Figure 2B). For the mutant p.Ala335LeufsTer20 allele, the expasy translation analysis proposed a 335 amino acid reading frameshift due to a premature stop codon, which resulted in a truncated TYR protein instead of 529 residues in normal protein (Figure 2C). The comparison between WT and mutant protein was made directly, which demonstrated that the truncated TYR protein for the p.Arg278Ter mutant showed loss of significant secondary structures and also showed conformational changes, which impacted the altered quaternary structures of the protein. Altogether, the reported pathogenic mutants (compound heterozygous) consequently suggested the defective synthesis of tyrosinase protein with a reduced enzymatic function that leads to the OCA1 phenotype (Figure 2A–C).

**Scheme 1:**
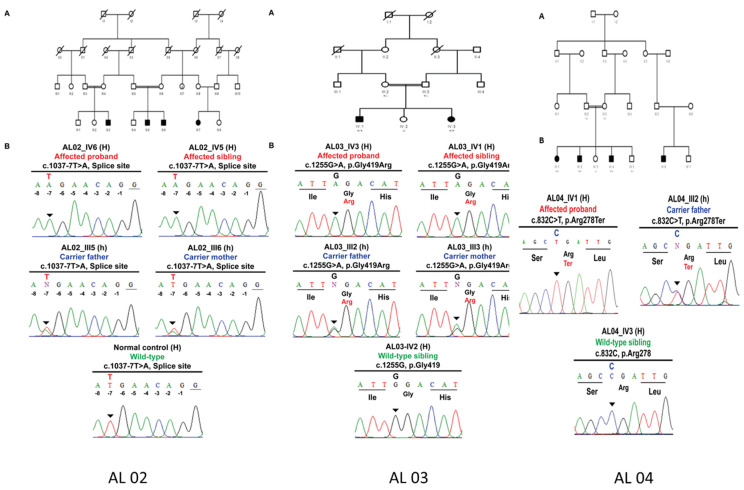
Homozygous splice site *TYR* c.1037-7T>A variant in AL02 family manifesting albinism. (**A**) Pedigree structure of AL02 family. (**B**) Sanger sequencing electropherograms reflecting familial segregation of *TYR* c.1037-7T>A pathogenic variant in AL02 family. Homozygous missense p.Gly419Arg variant in AL03 family manifesting albinism. (**A**) Pedigree structure of AL03 family. (**B**) Sanger sequencing electropherograms reflecting familial segregation of *TYR* p.Gly419Arg pathogenic variant in AL03 family. Homozygous nonsense *TYR* p.Arg278Ter variant in AL04 family manifesting albinism. (**A**) Pedigree structure of AL04 family. (**B**) Sanger sequencing electropherograms reflecting familial segregation of *TYR* p.Arg278Ter pathogenic variants in AL04 family.

In families AL02, AL03, and AL04, previously reported pathogenic variants including a splice-site error (c.1037-7T>A) and a missense pathogenic variant (c.1255G>A; p.Gly419Arg) in exon 4 and another non-sense pathogenic variant (c.832C>T; p.Arg278Ter) in exon 2 were identified, respectively, in the *TYR* gene (Scheme 1 and Table 2).

#### 3.2.2. Previous Pathogenic Variants in *OCA2*

**Scheme 2:**
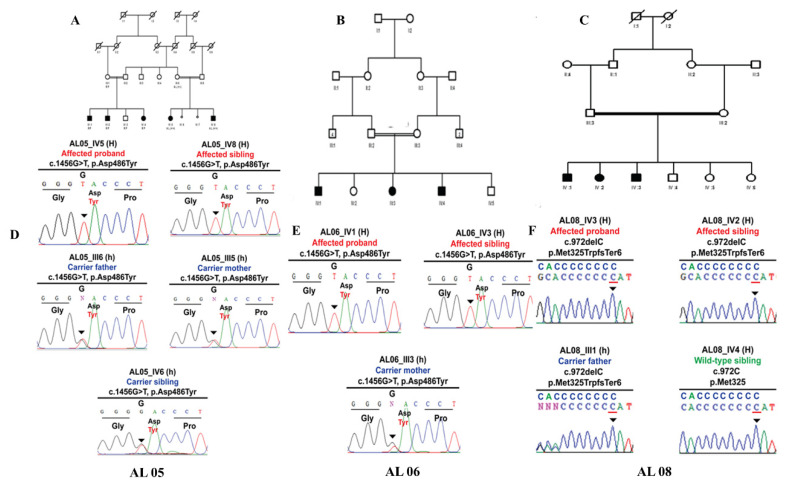
Homozygous Missense *OCA2* p.Asp486Tyr variant in AL05 family manifesting albinism (**A**–**F**). (**A**) Pedigree structure of AL05 family. (**D**) Sanger sequencing electropherograms reflecting familial segregation of OCA2 p.Asp486Tyr pathogenic variant in AL05 family. Homozygous Missense *OCA2* p.Asp486Tyr variant in AL06 family manifesting albinism. (**E**) Pedigree structure of AL06 family. (**B**) Sanger sequencing electropherograms reflecting familial segregation of *OCA2* p.Asp486Tyr pathogenic variant in AL06 family. Homozygous frameshift *HPS-1* p.Met325TrpfsTer6 variant in AL08 family manifesting albinism. (**C**) Pedigree structure of AL08 family. (**F**) Sanger sequencing electropherograms reflecting familial segregation of *HPS-1* p.Met325TrpfsTer6 pathogenic variant in AL01 family.

Family AL05 and AL06 were identified for known homozygous c.1456G>T; p.Asp486Tyr pathogenic variant in exon 13 of the *OCA2* gene. The homozygous pattern of mutants carries T/T allele at position c.1456G>T, whereas the heterozygous carrier showed a T/G allele pattern at the same position Scheme 2 and Table 2.

#### 3.2.3. Novel and Known HPS-1 Pathogenic Variants

**Figure 3:**
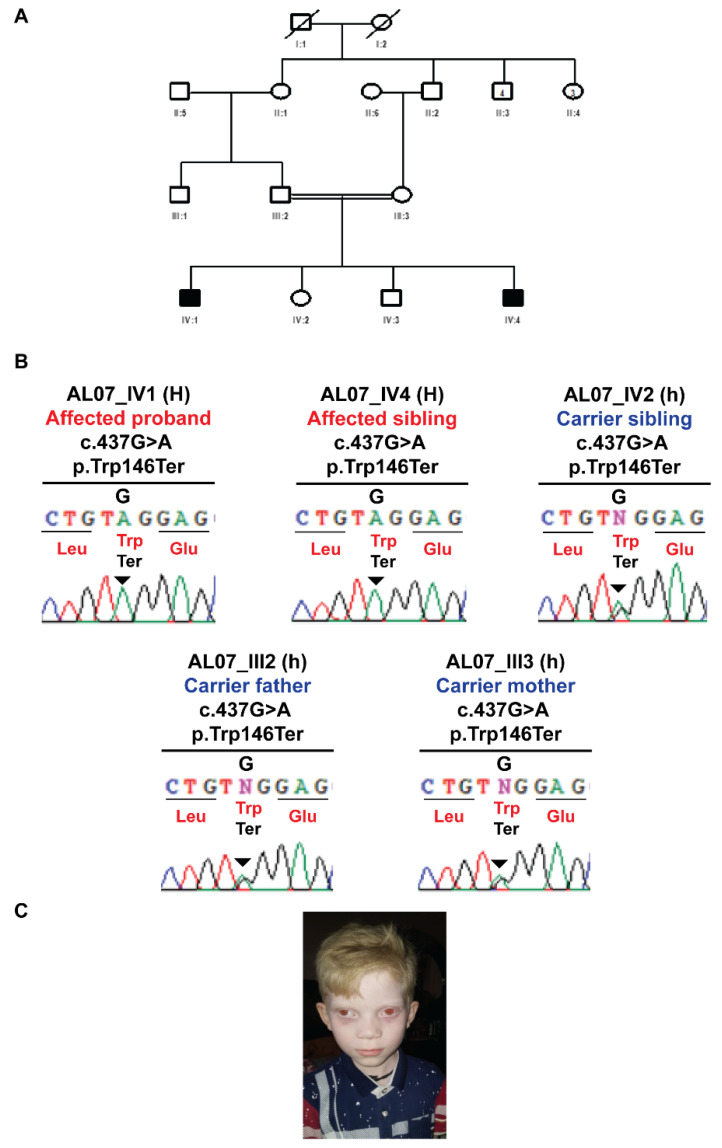
Homozygous nonsense *HPS1* p.Trp146Ter variant in AL07 family manifesting albinism. (**A**) Pedigree structure of AL07 family. (**B**) Sanger sequencing electropherograms reflecting the familial segregation of *HPS1* p.Trp146Ter novel pathogenic variant in AL07 family. (**C**) Clinical image depicting clinical features of albinism in pro-band IV-1 of family AL07.

In AL07, both affected individuals presented with all the cardinal features of nsOCA except eye color, but this family was not linked to any known gene of nsOCA. TruSight exome sequencing of one affected child revealed that it was linked to the Hermansky–Pudlak Syndrome 1 (*HPS1*) gene. Co-segregation analysis identified a novel nonsense pathogenic variant in family AL07; *HPS-1* c.437G>A; p.Trp146Ter. The heterozygous carrier parents had a G/A change, ehereas homozygous affected individuals had A/A instead of WT G/G, which resulted in the formation of a termination codon causing the premature termination of the protein (Figure 3 and Table 2).

In family AL08, the combination of TruSight exome sequencing and Sanger sequencing found the known frame-shift pathogenic variant in *HPS-1*; c.972delC; p.Met325TrpfsTer6 due to deletion of one base. The heterozygous carrier mother had a C/del change, whereas homozygous-affected individuals had del/del instead of WT C/C. This sequence change creates a premature translational stop signal (p.Met325TrpfsTer6) in the *HPS-1* gene (Scheme 2 and Table 2). It is expected to result in an absent or disrupted protein product. This variant is present in normal population databases (rs281865082, gnomAD 0.0001%) but not reported in a homozygous form.

## 4. Discussion

In this study, pathogenic variants of *TYR*, *OCA2*, and *HPS1* genes were detected in OCA families of Pakistani origin. Molecular genetic studies in eight albinism families detected novel and previously known pathogenic variants in different OCA genes. *TYR* pathogenic variants reported in the present study have also been investigated widely in various ethnic/language groups. In our study, known pathogenic variants c.1255G>A, c.832C>T, and 1037-7T>A of *TYR*, were found in three OCA families. To date, more than 600 pathogenic variants are linked to the *TYR* gene, and some pathogenic variants are very common, with high frequency in different populations (HGMD Professional, 2020). Pakistan is one of the leading countries in reported genetic disorders, and more than 200 OCA families have been reported from different research groups.

The most common pathogenic variant of the *TYR* gene reported in Pakistani OCA families, with a frequency of about 23%, is c.1255G>A; p.Gly419Arg and is predominantly found in the Punjabi-speaking language group. On the other hand, OCA families of other language groups, including Saraiki, Sindhi, Balochi, and Kashmiri, are reported for this common pathogenic variant [1,2,8,24,25,26,27]. The c.832C>T; p.Arg278Ter pathogenic variant is the second most frequent pathogenic variant, representing about 21% of total reported pathogenic variants, and it is also documented in the current study [23,25]. In addition, a previous splice-site pathogenic variant c.1037-7T>A has been reported in heterogeneous groups of Pakistan ancestry [2,24,25,27], and in the current study.

In Pakistan, the overall prevalence of pathogenic variant associated with *TYR* is nearly 44%, compared to the rate in the European population, where it is 46%. The reports of *TYR* pathogenic variants from other Asian groups are variable, but it is about 70% in a regional group of the Chinese population, showing remarkable heterogeneity [23,28]. A report from an Indian ethnic group showed a 60% prevalence of *TYR* pathogenic variants in patients with OCA features [29,30]. In addition, in other populations, including American, Korean, Japanese, and Italian people, the higher rate of *TYR* pathogenic variants is the main cause of the OCA phenotype [6].

In the Pakistani population, *OCA2* is the second causative gene of *OCA,* and 24% of diverse and frequently recurrent pathogenic variants are reported in this gene. In the present study, we identified one known c.1456G>T; p.Asp486Tyr pathogenic variant in two OCA families. Previously, this pathogenic variant was identified in five OCA families of the Pakistan region. Its frequency is almost 22.4%, making it one of the common alterations of the *OCA2* gene in the current study and in [2,8,23]. Interestingly, in the present study, an *OCA2* pathogenic variant was detected in two families belonging to the Dogar caste; one residing in Burewala, and the other was in Kasur. They were not distant relatives but might have had some common ancestors who harbored this particular pathogenic variant among the generations. Different in silico tools predicted this pathogenic variant as pathogenic. The residue was found in the conserved region when compared in different species orthologs of OCA2. It was anticipated that the biological action of the subsequent protein would be abnormal.

HPS subtype identification is important for accurate diagnosis, treatment options, clinical management, and prognosis. For example, BLOC-2 deficiency causes a milder type of phenotype with no life-threatening traits, and AP-3 defects result in immunodeficiency, whereas *BLOC-3* and *AP-3* pathogenic variants cause severe pulmonary fibrosis.

In addition, the current study investigated the involvement of the syndromic gene or genes in OCA families. In this study, one novel pathogenic variant, *HPS-1* c.437G>A; p.Trp146Ter, and one known pathogenic variant, p.Met325TrpfsTer6, have been identified in the *HPS-1* gene. *HPS-1*, the most common pathogenic variant of HPS, has been identified in individuals of different ethnicities all over the world. HPS-1 and HPS-4 manifest the most severe phenotypes. HPS-1-affected individuals are presented with more severe ocular findings than in other subtypes, and cutaneous albinism is more prominent with increased hypopigmentation of hair and skin [10]. The known frameshift pathogenic variant in exon 11 of *HPS-1*, p.Met325TrpfsTer6, present in one family, has also been described in different ethnicities, for example, in Japanese, Chinese, African American, Mexican, and Northern European populations. More than 76 HPS-1 pathogenic variants including 15 (20%) missense, 11 (14.5%) nonsense, 11 (14.5%) splice site, 20 (26%) frameshift, and 18 (24%) insertions and/or deletions pathogenic variants have been described to date [10,31,32,33,34,35].

In a previous study, it was noted that *HPS-1* pathogenic variants in mice appeared to have only a slight effect on bleeding and pigmentation for unknown reasons [36]. The same was observed in two families in this study with novel and known *HPS-1* pathogenic variants, as they did not show any bleeding tendency. According to Huizing et al., 2020, some HPS-1 affected individuals develop granulomatous colitis, most of them develop pulmonary fibrosis by middle age, and most of the affected females have menorrhagia [10], whereas HPS-1 affected cases in this study did not show any such symptoms. Phenotypically, we cannot distinguish between syndromic and non-syndromic OCA, so genetic testing is the gold standard for the exact diagnosis of such conditions.

Our study identified the pathogenic variants in *TYR*, *OCA2*, and *HSP-1* associated with the OCA phenotype. The results showed two novel pathogenic variants in *TYR* and *HSP-1* in two OCA families. In the other six families, the previously known pathogenic variants were identified in *TYR*, *OCA2*, and *HSP-1*. It is suggested that exploring the causative genes for OCA may expand the understanding of the molecular mechanism of the disease in specific ethnic groups, helping the community in genetic counseling. It may also be helpful in evolving the gene panels for accurate, cost-effective diagnosis of the most common OCA pathogenic variants present in the community that may further pave the pathway towards the treatment of such inherited diseases in future.

## Figures and Tables

**Table 1:** Physical and clinical features observed in OCA families.

Parameters		AL 01 III:5	AL 02 IV:6	AL 03 IV:3	AL 04 IV:1	AL 05 IV:5	AL 06 IV:1	AL 07 IV:1	AL 08 IV:3
Gender/Age (years)		Male/28	Male/21	Female/35	Female/15	Female/08	Male/18	Male/12	Male/16
Caste/Region		Mayo/Lahore	Cheema/Sargodha	Rajpoot/Rawalpindi	Arain/Sheikhupora	Dogar/Burewala	Dogar/Kasur	Bhatti Rajpoot/Lahore	Bukhari Sayyed/RYK
Hair colour		white	Light brown	White	White	Golden	Golden brown	Golden blond	Brown (dyed black)
Skin color		white	white	Reddish white	white	Reddish white	Reddish white	Reddish white	Reddish white
Skin rashes		On sunlight exposure	Not present	On sunlight exposure	On sunlight exposure	On sunlight exposure	On sunlight exposure	On sunlight exposure	Severe, without sunlight exposure
Iris color		Dark grey	Light brown	Light grey	Light grey	Greyish blue	Light Grey	Brown	Light grey
Visual acuity (BCVA LogMar)	R eye	0.9	NA	1.0	0.8	0.6	0.6	0.5	0.7
	L eye	0.9	NA	0.9	0.8	0.7	0.7	0.5	0.7
Refractive error	R	+3.0 × 170°	NA	8.0/+1 × 95°	+3.0/+1.5 × 85°	+4.5/−75°	+5.5/−4 × 166°	+4.5/−3 × 110°	+5.5
	L	+3.5 × 95°	NA	+8.0/+2 × 100°	+2.5/+4.5 × 100°	+5.5/+0.5 × 55°	+6.0/4.5 × 175°	+5.5/2.5 × 130°	+5.5/+0.5 × 55°
Photophobia		Present	Present	Present	Present	Present	Present	Present	Present
Nystagmus		Not Present	Present	Present	Present	Present	Present	Present	Present
Foveal hypoplasia		Present	Present	Present	Present	Present	Present	Present	Present
Fundus		Albiniotic	albiniotic	albiniotic	albiniotic	albiniotic	albiniotic	albiniotic	Albiniotic

Parameters; physical and clinical examination variables, AL; albinism famliy number.

**Table 2:** Novel and reported *TYR*, *OCA2* and *HPS-1* variants identified in albinism families in this study.

Families/Affected Individual	AL 01 III:5	AL 02 IV:6	AL 03 IV:3	AL 04 IV:1	AL 05 IV:5	AL 06 IV:1	AL 07 IV:1	AL 08 IV:3
Causative Gene	*TYR*	*TYR*	*TYR*	*TYR*	*OCA2*	*OCA2*	*HPS1*	*HPS1*
Pathogenic variant	c.832C>T; c.1002delA	c.1037-7T>A	c.1255G>A	c.832C>T	c.1456G>T	c.1456G>T	c.437G>A	c.972delC
Protein position	p.Arg278Ter p.Ala335LeufsTer20	----	p.Gly419Arg	p.Arg278Ter	p.Asp486Tyr	p.Asp486Tyr	p.Trp146Ter	p.Met325TrpfsTer6
Status/Type of pathogenic variant	Compound Heterozygous/Nonsense; frameshift	Homozygous/Splice site	Homozygous/Missense	Homozygous/Nonsense	Homozygous/Missense	Homozygous/Missense	Homozygous/Nonsense	Homozygous/Frameshift
Previously reported	Novel (this study)	Yes [2,20]	Yes [21]	Yes [22]	Yes [2,23]	Yes [2,23]	Novel (this study)	Yes [10,13]
SIFT	--------	--------	Damaging	--------	Damaging	Damaging	--------	--------
Pathogenic variant Taster	Disease causing; Disease causing	Disease causing	Disease causing	Disease causing	Disease causing	Disease causing	Disease causing	Disease causing
PolyPhen-2	--------	--------	Probably damaging	--------	Probably damaging	Probably damaging	--------	--------
Evolutionary conservation	--------	--------	*Ciona intestinalis*	--------	*Drosophila melanogaster*	*Drosophila melanogaster*	--------	--------
gnomAD (All)	0/48/282,454; ------	1/242/280,938	0/17/281,824	0/48/282,454	0/6/251,494	0/6/251,494	--------	0/28/175,586
gnomAD (South Asians)	0/39/30,606;---------	1/155/10,330	0/12/30,606	0/39/30,606	0/6/30,616	0/6/30,616	----------	0/15/17,454

## Data Availability

Data available within the article or its Supplementary Materials. The authors confirm that the data supporting the findings of this study are available within the article [and/or] its Supplementary Materials.
